# Comparative Efficacy of High-Dose Dexamethasone Versus Methylprednisolone in Coronavirus Disease 2019 (COVID-19)-Associated Acute Respiratory Distress Syndrome

**DOI:** 10.7759/cureus.55725

**Published:** 2024-03-07

**Authors:** Pedja Kovacevic, Jovana Malic, Tijana Kovacevic, Sasa Dragic, Biljana Zlojutro, Milka Jandric, Danica Momcicevic, Branka Cancarevic-Djajic, Ranko Skrbic, Zeeshan M Rizwan

**Affiliations:** 1 Critical Care Medicine, Medical Intensive Care Unit, University Clinical Centre of the Republic of Srpska, Banja Luka, BIH; 2 Hospital-Based Medicine, Faculty of Medicine, University of Banja Luka, Banja Luka, BIH; 3 Critical Care Medicine, Mayo Clinic Alix School of Medicine, Rochester, USA

**Keywords:** ards, covid-19, dexamethasone, methylprednisolone, outcome

## Abstract

Objective: This retrospective (matched paired) clinical trial aimed to compare the efficacy of dexamethasone vs. methylprednisolone at equipotent (high) doses in patients with coronavirus disease 2019 (COVID-19)-associated acute respiratory distress syndrome (ARDS).

Methods: A total of 347 patients with moderate and severe COVID-19-associated ARDS were administered either a high (equipotent) dose of dexamethasone (32 mg) or methylprednisolone (180 mg) for a duration of up to 10 days. All participants received the standard of care for critically ill COVID-19 patients.

Results: The primary outcomes included length of stay in the ICU, ICU mortality, and discharge from the hospital.* *Based on the obtained results, a tendency towards more favorable clinical outcomes concerning the length of stay in the ICU (in the group of patients treated with non-invasive mechanical ventilation (NIV), p<0.05), ICU mortality, and discharge from the hospital (in the group of patients who were intubated, p<0.05) in patients receiving the high dose of dexamethasone compared to those receiving methylprednisolone was observed.

Conclusion: It appears that severe cases of COVID-19, especially intubated ones, treated with high doses of dexamethasone have a more favorable clinical outcome than the use of equipotent doses of methylprednisolone. However, larger multicenter studies are needed to validate our observations.

## Introduction

Coronavirus disease 2019 (COVID-19), a respiratory syndrome caused by severe acute respiratory syndrome coronavirus 2 (SARS-CoV-2), has had a significant impact on global health and affected millions of people worldwide. Efforts to control the spread of COVID-19 highlight the importance of continued research and public health interventions [1], which can have a significant impact on global healthcare systems. The clinical presentation of these patients can vary greatly. Most of them have very mild or asymptomatic forms of COVID-19, but a smaller percentage may develop severe forms of pneumonia, complicated with acute respiratory distress syndrome (ARDS) [2]. ARDS is associated with a high mortality rate and is presented with hypoxemia and tachypnoea (respiratory distress), with chest X-ray revealing bilateral infiltrates. The diagnosis of ARDS in COVID-19 patients follows the well-established Berlin definition of ARDS [3,4]. This syndrome and the problems related to its treatment are particularly manifested in low-resource settings, where the mortality rate for intubated patients can be as high as 80% [5]. Today, it is known that the dysregulation and excessive response of the immune system (cytokine storm) is one of the key links in the chain of pathophysiological events in patients who develop ARDS associated with COVID-19 [6,7]. Considering all of the above, it has been hypothesized that drugs with anti-inflammatory effects may contribute to a better outcome in patients suffering from ARDS associated with COVID-19 [8]. Among the many observed and tested anti-inflammatory drugs, corticosteroids have gained much attention worldwide due to their accessibility and affordability [9]. While corticosteroids are recommended, specifics such as the preferred corticosteroids, optimal dose, and treatment duration remain undetermined [9]. The clinical study called "The Recovery" was the first to provide data showing that patients with severe forms of COVID-19 (requiring oxygen therapy or mechanical ventilation) who were administered dexamethasone (DXM) experienced a decreased mortality rate [10]. Most guidelines for COVID-19 treatment recommend DXM as the first choice at a dose of 6 mg once daily for up to 10 days. Furthermore, other corticosteroids, such as methylprednisolone (MTP) and hydrocortisone at equipotent doses, are considered alternatives in case of DXM shortage [11,12]. The dosing regimens employed in major randomized controlled trials for corticosteroids in critical COVID‐19 pneumonia are typically in the approximate range of 0.5-2 mg/kg/day of MTP. In contrast, the highest investigated dose of DXM has been 20 mg daily, equivalent to 107 mg of MTP [13]. Some studies suggest that MTP is more effective than DXM. However, it is important to acknowledge that potential biases, such as inconsistent dosing, may obscure these findings [14-18]. The primary outcome of this research was to assess and compare the individual outcome of COVID-19-associated ARDS patients receiving equipotent doses of either high-dose DXM or MTP as anti-inflammatory agents.

## Materials and methods

Study designs

A matched pairs, retrospective observational study was created in the Medical Intensive Care Unit (MICU) of the University Clinical Centre of the Republic of Srpska (UCC RS) in Banja Luka, Bosnia and Herzegovina, between April 1, 2020, and January 1, 2022. This MICU was established over the last 15 years with support from critical care experts from Europe and the United States [19,20]. Currently, this is the only multidisciplinary MICU in Bosnia and Herzegovina. Approval from the Ethics Committee and signed informed consent were not necessary, as this represents an established local protocol for the treatment of critically ill patients with COVID-19-associated ARDS.

Study population (demographic, clinical, and laboratory characteristics)

The study included all patients who developed COVID-19-related ARDS over the age of 18 who were admitted to the MICU at the UCC RS and met the inclusion criteria. Nasopharyngeal swabs and respiratory secretions were analyzed using reverse transcription polymerase chain reaction (RT-PCR). Inclusion criteria were as follows: age over 18 years, confirmed diagnosis of COVID-19 infection through positive RT-PCR test, presence of radiologic signs of ARDS (bilateral pulmonary infiltrates), and classification as mild, moderate, or severe ARDS according to the Berlin criteria [4]. Patients who met one or more of the following exclusion criteria were not included in the study: fatal outcome or significant recovery within 48 hours of randomized allocation, end-stage disease, chronic corticosteroid treatment, immunosuppression or immunosuppressive therapy, known hypersensitivity to corticosteroids, receiving both DXM and MTP, and under palliative care. Patients were categorized into two groups (A and B) depending on the type of ventilatory support (from the lowest to the highest level of ventilatory support) and the severity of ARDS, as shown in Table 1.

**Table 1:** Two groups of patients based on the severity of ARDS and mode of respiratory support. ARDS: acute respiratory distress syndrome; NIV: non-invasive ventilation; vvECMO: venovenous extracorporeal membrane oxygenation

Group of patients	Mode of respiratory support/severity of ARDS
Group A	Patients who required NIV (including patients with a P/F ratio >100 and ≤200; moderate ARDS according to the Berlin criteria for ARDS)
Group B	Patients who required invasive mechanical ventilation via an endotracheal tube and vvECMO for those with a P/F ratio <100; patients with severe ARDS according to the Berlin criteria for ARDS

Demographic characteristics included age and sex. Clinical characteristics included comorbidities and the final outcome of treatment, length of ICU stay (in days), Simplified Acute Physiology Score (SAPS) score, Sequential Organ Failure Assessment (SOFA) score, and Acute Physiology and Chronic Health Evaluation (APACHE) II score, type of steroids, and administration of tocilizumab. Various laboratory tests and assessments were carried out on admission: white blood cell (WBC) count, neutrophil-to-lymphocyte ratio (NLR), C-reactive protein (CRP), lactate dehydrogenase (LDH), ferritin, D-dimer levels, acute kidney injury (AKI) assessment, continuous renal replacement therapy (CRRT) evaluation, and presence of pneumothorax.

Intervention (local protocol for corticosteroid treatment)

The local protocol for the treatment of critically ill COVID-19-associated ARDS in the MICU of the UCC RS primarily involves the administration of MTP at a dosage of 2 mg/kg/day (160 mg intravenously). This treatment is usually continued for up to 10 days, with the total daily dose divided into two separate administrations. Throughout the pandemic, the hospital pharmacy experienced multiple drug supply shortages, resulting in DXM being used for up to 10 days (32 mg/day intravenously, the total daily dose divided into two separate administrations) at an equipotent dose of MTP according to the local corticosteroid treatment protocol. The dosage of MTP (2 mg/kg/day) was determined based on the institution's experience in managing pneumonia, organized pneumonia, and ARDS caused by various underlying factors. All critically ill patients received standard treatment for COVID-19-associated ARDS according to the local hospital protocol, derived from guidelines provided by the local Ministry of Health and international recommendations. Data were systematically collected from patients' medical records using a standardized data collection form by a senior medical resident. After verification and confirmation by the attending physician, the collected data was then entered into an electronic database.

Statistical analysis

IBM SPSS Statistics for Windows, Version 26.0 (Released 2019; IBM Corp., Armonk, New York, United States) was used to analyze the data in the study. The Kolmogorov-Smirnov and Shapiro-Wilk tests were used to test the normality of data distribution. Continuous variables were expressed as median (interquartile range (IQR)), and categorical variables were expressed as numbers and proportions. The Mann-Whitney U and Kruskal-Wallis tests were used for comparing continuous variables, whereas Fisher's exact test and Pearson's tests were used for comparing categorical data between the groups. We considered p<0.05 statistically significant. A robust Cox regression model analysis was carried out to identify the predictor variables that explain the hazard ratio (HR) with 95% CI, avoiding a possible interference of outlier observations in the partial likelihood estimation.

## Results

**Figure 1:**
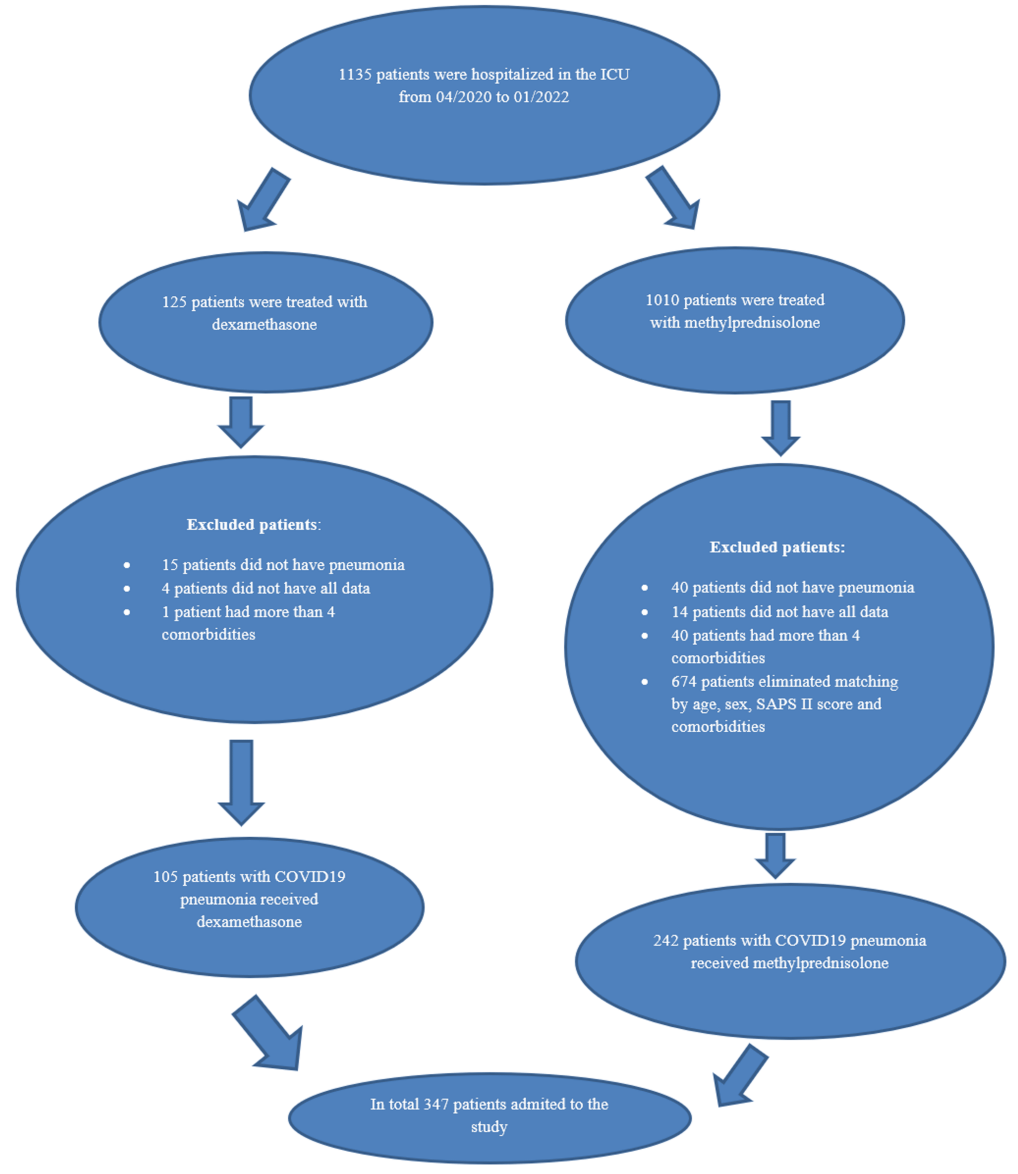
Flowchart of patient selection and follow-up.

A total of 1135 COVID-19 cases were admitted to the MICU over 21 months. Out of these cases, 347 patients with COVID-19-associated ARDS were ultimately included in the data analysis. Among them, 109 patients received DXM, while 258 patients were administered MTP (Figure 1). On MICU day 1, no statistically significant differences were found between the two patient groups concerning age, sex, comorbidities (except smoking status), CRP, LDH, ferritin, and D-dimer levels. Likewise, in terms of patient severity (as indicated by SAPS II, SOFA, and APACHE II scores) and final treatment outcomes, the groups exhibited homogeneity (Table 2). In all observed subgroups of patients (groups A and B), the number of patients receiving DXM and MTP showed no statistically significant differences (p=0.426); the groups were homogeneous.

**Table 2:** Demographic and clinical characteristics and final outcome of all observed patients with COVID-19-associated ARDS for the treatment group. DXM: dexamethasone; MTP: methylprednisolone; SOFA: Sequential Organ Failure Assessment; SAPS II: Simplified Acute Physiology Score II; APACHE II: Acute Physiology and Chronic Health Evaluation II; COVID-19: coronavirus disease 2019; ARDS: acute respiratory distress syndrome

Variable	Treatment		Mann-Whitney U test	Chi-squared test	Total patients (n=347)
	DXM (n=105)	MTP (n=242)	P-value	P-value	
Age (years)	62 (17)	64 (14)	0.390	-	63 (14)
Sex					
Male	78 (30.7%)	176 (69.3%)	-	0.763	254 (73.2%)
Female	27 (29%)	66 (71%)	-		93 (26.8%)
Comorbidities					
Diabetes mellitus	29 (33.3%)	58 (66.7%)	-	0.471	87 (25.1%)
Chronic heart disease	40 (26.8%)	109 (73.2%)	-	0.230	149 (42.9%)
Chronic lung disease	7 (21.9%)	25 (78.1%)	-	0.279	32 (9.2%)
Chronic kidney disease	4 (30.8%)	9 (69.2%)	-	0.967	13 (3.7%)
Chronic liver disease	0	4 (100%)	-	0.185	4 (1.2%)
Chronic rheumatoid disease	0	7 (100%)	-	0.780	7 (2%)
Hypothyreosis	9 (50%)	9 (50%)	-	0.061	18 (5.2%)
Obesity	15 (42.9%)	20 (57.1%)	-	0.087	35 (10.1%)
Smokers	15 (55.6%)	12 (44.4%)	-	0.003	27 (7.8%)
Score					
SOFA	4 (5)	4 (5)	0.900	-	4 (3)
SAPS II	22 (14)	22 (13)	0.512	-	22 (14)
APACHE II	7 (10)	7 (10)	0.906	-	7 (10)
Outcome					
Deceased in ICU	68 (27.6%)	178 (72.4%)	-	0.098	246 (70.9%)
Deceased on the ward	6 (50%)	6 (50%)	-	0.130	12 (3.5%)
Discharged home	31 (36.5%)	58 (65.9%)	-	0.276	89 (25.64%)

In patient group A (consisting of patients treated with NIV only), no statistically significant differences were observed in all measured parameters, including outcomes, when evaluating the impact of the different types of corticosteroid therapy (DXM and MTP), except for the length of ICU stay. The length of ICU stay was shorter in patient group B treated with DXM, with a median of six days (3-10), compared to MTP with a median of 7.5 days (5-13.75) (p-value<0.039). Refer to Table 3 for details.

**Table 3:** Clinical characteristics and final outcomes of patients treated with NIV (group A) using two types of corticosteroids. NIV: non-invasive mechanical ventilation; DXM: dexamethasone; MTP: methylprednisolone; CRP: C-reactive protein; LDH: lactate dehydrogenase; CRRT: continuous renal replacement therapy

Ventilation with NIV	Treatment		Mann-Whitney U test	Chi-squared test	Total patients (n=79)
	DXM (n=27)	MTP (n=52)	P-value	P-value	
Leukocytes	10.99 (5.92)	11.28 (4.86)	0.877	-	11.19 (5.24)
Lymphocyte/granulocyte ratio	12.17 (10.57)	13.38 (9.76)	0.590	-	12.70 (10)
CRP	105 (130.7)	64.05 (129.95)	0.189	-	70.20 (136)
LDH	455.5 (305.75)	512 (248)	0.464	-	481 (260)
Ferritin	1370 (606.5)	928 (771.75)	0.990	-	963 (852)
D-dimer	1.58 (4.82)	2.28 (8.2)	0.560	-	2.07 (7.3)
Pneumothorax	0	0	-	-	0
Acute kidney injury	2 (40.0%)	3 (60.0%)	-	0.777	5 (6.33%)
CRRT	0	0	-	-	0
Tocilizumab	1 (12.5%)	7 (87.5%)	-	0.173	8 (10.13%)
Length of stay in ICU (in days)	6 (7)	7.50 (8.75)	0.039	-	7 (8)
Outcome					
Deceased in ICU	5 (18.5%)	4 (7.7%)	0.151	-	9 (11.4%)
Deceased on the ward	3 (11.1%)	2 (3.8%)	0.208	-	5 (6.33%)
Discharged home	19 (70.4%)	45 (86.5%)	0.082	-	64 (81.01%)

Through a proportional hazard model, the influence of the variables on the hazard rate function of the response variable "NIV patients ICU days/time to recovery" was tested. The robust Cox model showed that only the variables "AKI (acute kidney injury)" and "Age" were significant for modeling the hazard rate function (Table 4).

**Table 4:** Length of stay in ICU/time to recovery performance analysis: Cox models (NIV patients' ICU days). OR: odds ratio; AKI: acute kidney injury; DXM: dexamethasone; MTP: methylprednisolone; NIV: non-invasive ventilation

Explanatory variable	OR	P-value
Treatment (1-MTP vs. 2-DXM)	1.299 (0.773-2.182)	0.324
AKI	3.398 (1.026-11.25)	0.045
Age (years)	0.976 (0.955-0.997)	0.025
Sex (men)	1.237 (0.687-2.228)	0.478

Patients with AKI had a 3.398-fold higher risk of longer ICU stays compared to those without AKI (p=0.045). Age was also found to be a significant factor, suggesting that risk changes proportionally with increasing age. Although patients receiving MTP stayed significantly longer in the ICU than patients treated with DXM in univariate analysis, this difference becomes less significant in the Cox model when other factors influencing the length of stay in the ICU (AKI and age) were included.

**Figure 2:**
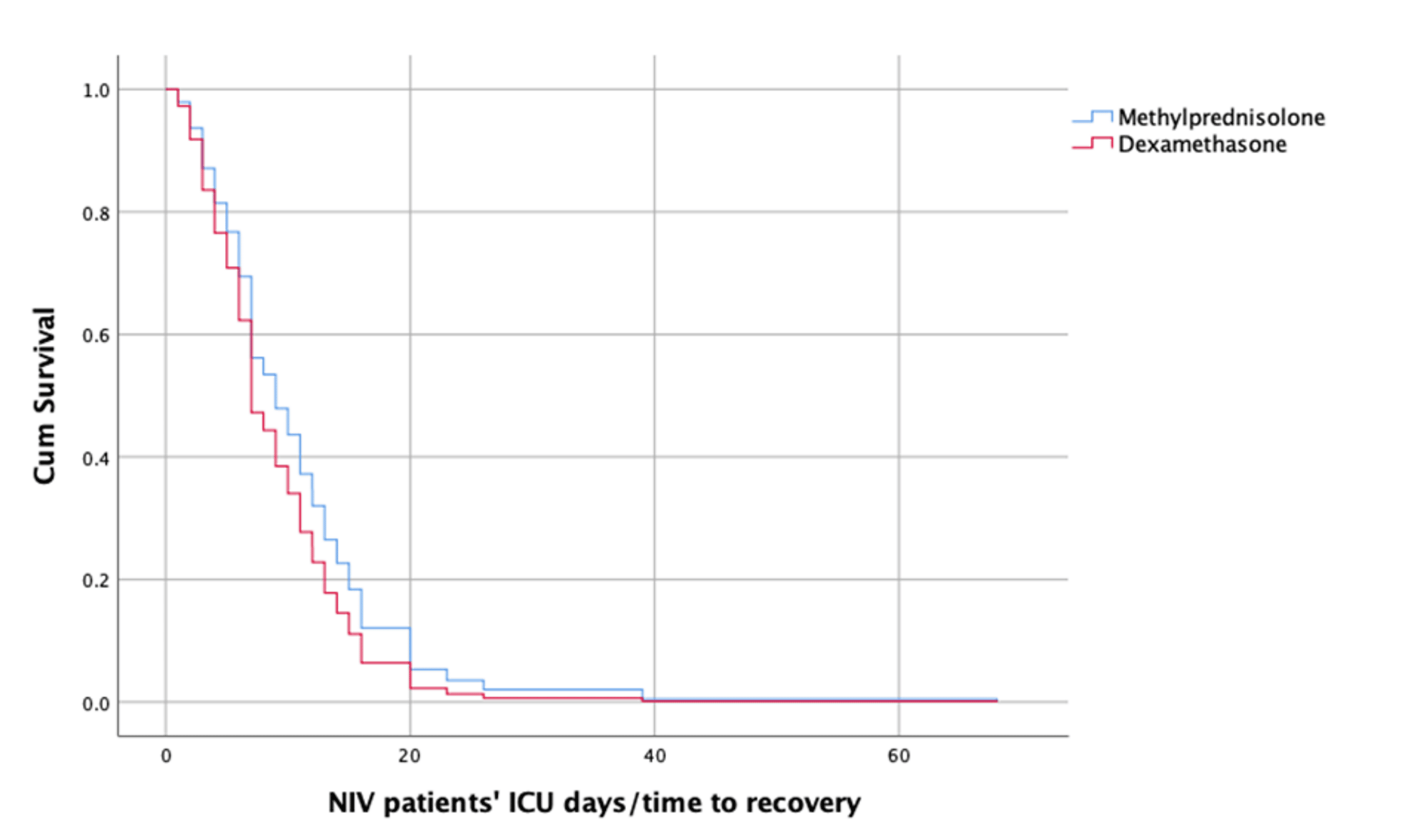
Survival function estimated by Cox model, for patients treated with NIV only, according to treatment received: MTP vs. DXM. DXM: dexamethasone; MTP: methylprednisolone; NIV: non-invasive ventilation

**Figure 3:**
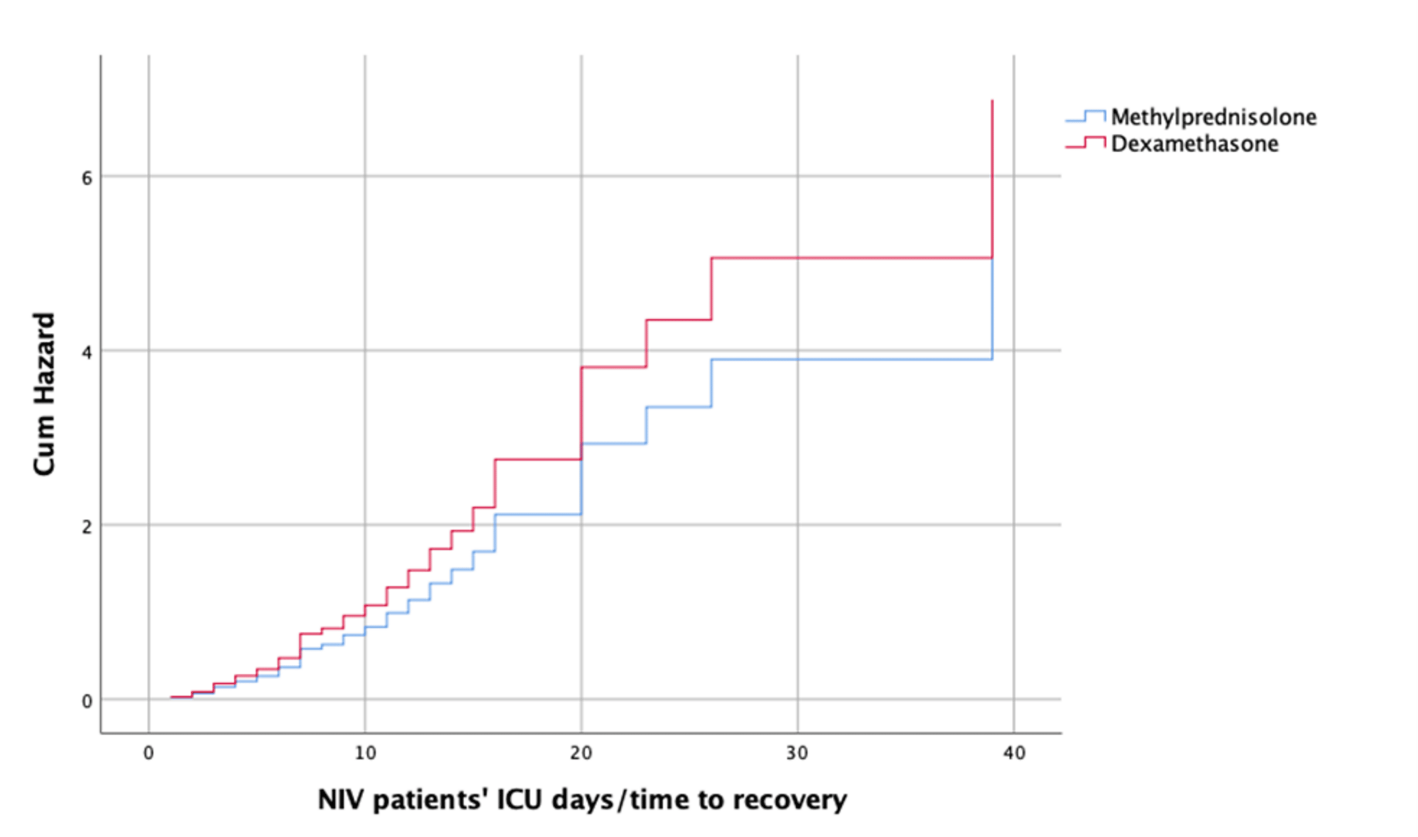
Cumulative hazard function estimated by Cox model, for patients treated with NIV only, according to the MTP vs. DXM treatment. DXM: dexamethasone; MTP: methylprednisolone; NIV: non-invasive ventilation

For the variable "Treatment," the estimated regression coefficient indicated that the treatment with DXM has the same effect as MTP on time to recovery (Figure 2 and Figure 3).

In patient group B (consisting of patients treated exclusively with invasive mechanical ventilation), no statistically significant differences were found in all measured parameters when evaluating the effects of the different types of corticosteroid therapy (DXM and MTP), with the exception of deaths in the ICU and discharges from hospital. The number of deceased patients in ICU was lower in patients treated with DXM, 63 patients (80.8%), compared to MTP, where 174 patients (91.6%) died (p-value<0.012). Discharge from the hospital showed statistical significance in the patient group treated with DXM, with 12 patients (15.4%), in comparison to patients treated with MTP, where nine patients (4.7%) were discharged from the hospital (p-value<0.03). Refer to Table 5 for more details.

**Table 5:** Clinical characteristics and final outcomes of patients treated with IMV (group B) using two types of corticosteroids. IMV: invasive mechanical ventilation; DXM: dexamethasone; MTP: methylprednisolone; CRP: C-reactive protein; LDH: lactate dehydrogenase; CRRT: continuous renal replacement therapy

Ventilation with IMV	Treatment		Mann-Whitney U test	Chi-squared test	Total patients (n=268)
	DXM (n=78)	MTP (n=190)	P-value	P-value	
Leukocytes	12.02 (8.38)	12.52 (8.2)	0.180	-	12.52 (7.92)
Lymphocyte/granulocyte ratio	14.25 (23.39)	16.26 (21.02)	0.251	-	15.90 (22.28)
CRP	113.50 (124.25)	80 (117.5)	0.117	-	94.35 (129.22)
LDH	541 (295)	590 (333.25)	0.387	-	572 (320.75)
Ferritin	1184 (1215)	1223 (907)	0.633	-	1215.50 (892.5)
D-dimer	2.01 (7.05)	2.57 (9.34)	0.284	-	2.44 (8.44)
Pneumothorax	6 (31.6%)	13 (68.4%)	-	0.805	19 (7.09%)
Acute kidney injury	31 (27.4%)	82 (72.6%)	-	0.607	113 (42.16%)
CRRT	15 (30%)	35 (70%)	-	0.877	50 (18.65%)
Tocilizumab	3 (37.5%)	5 (62.5%)	-	0.601	8 (2.98%)
Length of stay in ICU (in days)	10.5 (15)	8 (9.25)	0.057	-	9 (10)
Outcome					
Deceased in ICU	63 (80.8%)	174 (91.6%)	0.012	-	237 (88.43%)
Deceased in the ward	3 (3.8%)	4 (2.1%)	0.417	-	7 (2.61%)
Discharged home	12 (15.4%)	9 (4.7%)	0.030	-	21 (7.83%)

## Discussion

The main findings of this study highlight a notable decrease in the ICU mortality rate, along with a substantial increase in the number of patients who were discharged from the hospital. This positive outcome was observed in intubated patients with COVID-19-associated ARDS who were treated with high doses of DXM compared to patients treated with equipotent doses of MTP. Notably, to the best of our knowledge, this is the sole study that compares the outcomes of critically ill COVID-19 patients treated with high doses of DXM against equipotent doses of MTP on mechanical ventilation (Table 6) [21].

**Table 6:** Logistic regression analysis: association of type of treatment (MTP vs. DXM) with mortality in patients in group B (invasive mechanical ventilation) OR: odds ratio; AKI: acute kidney injury; DXM: dexamethasone; MTP: methylprednisolone

Explanatory variable	OR (95% CI)	P-value
Treatment (1-MTP, 2-DXM)	2.541 (1.161-5.564)	0.020
AKI	4.700 (1.704-12.962)	0.003
Age (years)	1.034 (0.995-1.073)	0.086
Sex (men)	1.001 (0.423-2.370)	0.998

Our findings demonstrate that high doses of DXM are associated with better outcomes in critically ill COVID-19 patients. During the early period of the COVID-19 pandemic, it became evident that the disease is mediated by the immune system [22,23]. Following this realization, the healthcare authorities of the Republic of Srpska (Bosnia and Herzegovina) issued recommendations for the treatment of the most severe forms of COVID-19, especially for COVID-19-associated ARDS [24]. The treatment guidelines for these patients included the administration of MTP at a dosage of 2 mg/kg/day, divided into two doses (160 mg). The rationale for choosing MTP as the preferred medication was related to the limited resources available in Bosnia and Herzegovina [19]. Given the constrained circumstances, the availability of DXM, as well as other immunosuppressive drugs such as tocilizumab, was restricted. The dosage of MTP was derived from previous recommendations (2 mg/kg/day) for the treatment of critically ill patients, individuals with ARDS, and those suffering from immunologically mediated pneumonia [25-27]. On the other hand, Chang et al. conducted a study (a systematic review and meta‑analysis) to evaluate the impact of (different types) corticosteroid treatment on the prognosis of patients with ARDS of various etiologies. The findings of the study demonstrated that the use of corticosteroids, particularly MTP, had a positive effect on the survival rate of patients with ARDS, regardless of the underlying cause. This suggests that treatment with corticosteroids may improve survival in patients with ARDS of different etiologies. The existing literature suggests that MTP is effective in severe COVID-19 patients, with a usual dosage range of 1-2 mg/kg/day [18,28-30]. On the other hand, there is a limited availability of comparative studies that assess the effectiveness of MTP versus DXM among mechanically ventilated COVID-19 patients. The majority of the existing studies do not compare equipotent dosages. The aforementioned studies most often compare MTP (1-2 mg/kg/day) with DXM (6 mg/day) or even disparate dosages like MTP (500 mg/day) with DXM (6 mg/day) [15,30]. Based on the evidence of the majority of analyzed randomized controlled trials, MTP, particularly at moderate to high doses, exhibits an advantage over DXM in the treatment of patients with severe COVID-19 [15,18,30]. A comparison of the final outcomes in mechanically ventilated COVID-19-associated ARDS cases treated with equipotent doses (high doses) of MTP and DXM has not been performed. Therefore, it is difficult to compare our results with those of the other authors. A very similar study, focusing on lower equipotent doses of corticosteroids (DXM 6 mg/day vs. MTP 32 mg/day vs. hydrocortisone 150 mg/day) in mechanically ventilated patients, aligns with our findings, highlighting the superiority of DXM [12,31]. An in-depth analysis of critically ill COVID-19 patients requiring different types of respiratory support (NIV and invasive mechanical ventilation) highlights the increased efficacy of high doses of DXM as the disease severity increases corroborating findings from other studies [32]. The optimal dosage of corticosteroids, particularly DXM, for critically ill COVID-19 patients remains a controversial and intriguing question [18,33,34]. Given the limitations, the results of this study should be interpreted with caution. The primary limitation is the small number of participants, which typically reduces the power of the study. Another important limitation is that the study was conducted at a single center, potentially limiting its generalizability to other centers. Finally, a possible limitation is the absence of a control group, which was not administered any corticosteroid, because of ethical reasons. The findings and their implications should be discussed in the broadest context possible. Future research directions can also be highlighted.

## Conclusions

The current data suggest that using DXM at an equipotent high dose may be more effective than MTP in determining the individual outcomes of critically ill COVID-19 patients, especially for those who require intubation. Patients who are on a lower level of respiratory support may require a lower equipotent dose of DXM. However, larger multicenter trials are needed to validate these findings.
